# Analysis of Cross-Reactive Neutralizing Antibodies in Human HFMD Serum with an EV71 Pseudovirus-Based Assay

**DOI:** 10.1371/journal.pone.0100545

**Published:** 2014-06-25

**Authors:** Huafei Zhang, Dong An, Wei Liu, Qunying Mao, Jun Jin, Lin Xu, Shiyang Sun, Liping Jiang, Xiaojun Li, Jie Shao, Hongxia Ma, Xueyong Huang, Shijie Guo, Haiying Chen, Tong Cheng, Lisheng Yang, Weiheng Su, Wei Kong, Zhenglun Liang, Chunlai Jiang

**Affiliations:** 1 School of Life Sciences, Jilin University, Changchun, P. R. China; 2 National Engineering Laboratory for AIDS Vaccine, Jilin University, Changchun, P. R. China; 3 National Institutes for Food and Drug Control, Beijing, P. R. China; 4 Henan Provincial Center for Disease Control and Prevention, Zhengzhou, P. R. China; 5 Department of Pediatrics, the First Hospital of Jilin University, Changchun, P. R. China; 6 National Institute of Diagnostics and Vaccine Development in Infectious Disease, School of Life Science, Xiamen University, Xiamen, P. R. China; 7 Key Laboratory for Molecular Enzymology & Engineering, Ministry of Education, Jilin University, Changchun, P. R. China; Johns Hopkins School of Public Health, United States of America

## Abstract

Hand, foot and mouth disease, associated with enterovirus 71 (EV71) infections, has recently become an important public health issue throughout the world. Serum neutralizing antibodies are major indicators of EV71 infection and protective immunity. However, the potential for cross-reactivity of neutralizing antibodies for different EV71 genotypes and subgenotypes is unclear. Here we measured the cross-reactive neutralizing antibody titers against EV71 of different genotypes or subgenotypes in sera collected from EV71-infected children and vaccine-inoculated children in a phase III clinical trial (ClinicalTrials.gov Identifier: NCT01636245) using a new pseudovirus-based neutralization assay. Antibodies induced by EV71-C4a were cross-reactive for different EV71 genotypes, demonstrating that C4a is a good candidate strain for an EV71 vaccine. Our study also demonstrated that this new assay is practical for analyses of clinical samples from epidemiological and vaccine studies.

## Introduction

Hand, foot and mouth disease (HFMD) is a common illness in children, particularly in those less than five years of age [1]. Since it was first reported in 1957, several large outbreaks of HFMD have been reported in eastern and southeastern Asian countries and regions. The earliest known case of HFMD in China was reported in Shanghai in 1981 and was followed by reports of HFMD in most Chinese provinces [1]. Since 2008, increasing numbers of HFMD cases have been reported by the Chinese Center for Disease Control and Prevention with 2,713 deaths until February 2014 in China (Chinese Center for Disease Control and Prevention website: http://www.chinacdc.cn/tjsj/fdcrbbg/). These increasingly large and severe HFMD epidemics are a major public health concern in mainland China [2].

Enterovirus 71 (EV71) is the major causative agent of HFMD [3]. Since EV71 was first identified in a child with neurological symptoms in California in 1969, EV71-associated outbreaks have been reported worldwide [4]. Infected children often present with fever for 3–4 days and develop vesicles on the hands, feet, elbows, knees and buccal mucosa. In most instances, this illness is mild and self-limiting. However, EV71 infection sometimes causes severe neurological disorders, such as aseptic meningitis, encephalitis, poliomyelitis-like paralysis and even death [5]–[7].

EV71 is a member of the *Human enterovirus A* (HEV-A) species, belonging to the genus *Enterovirus* in the family Picornaviridae [8]. It is a small, single-stranded, positive-sense RNA virus. EV71 has one serotype but can be classified into three main genogroups (A, B, and C) and 11 subgenotypes (A, B1–B5, and C1–C5) by analysis of the most variable capsid protein sequence (VP1) [9], [10]. The prototype BrCr strain, which is the solo member of group A, was first identified in California in 1970 [11]. The circulation of strains B1 and B2 were well-documented in the United States in the 1970s and 1980s [11]. Thereafter, predominant strains in Malaysia, Singapore and Western Australia were identified as subgenotypes B3 and B4 [12], whereas B5 strains were isolated from EV71 cases in Japan (2003), Taiwan (2003, 2007–2008) and Brunei (2006) [1]. Low-level circulation of subgenotype C1 virus was initially recorded sporadically in the 1980s, except for the major community outbreak in Sydney, Australia [13]. Large outbreaks of subgenotype C2 viruses were reported in Taiwan (1998) and Australia (1999), and it was also found in Japan in 1997–1999 and 2001–2002 [12], [14]. C3 was first isolated in Japan (1994) and subsequently in mainland China (1997). In Korea (2000), C3 was reported as the predominant strain in an EV71 outbreak [14], [15]. Since 1998, the C4 subgenotype was isolated from sporadic infections in mainland China and became the major causative agent of EV71 epidemics in recent years [16]. Furthermore, this subgroup has been reported in Japan, Vietnam and especially in Taiwan, where it was responsible for large outbreaks in 2004–2005, and then was replaced by subgenotypes B5 and C5 [17], [18]. Subgenotype C4 also has been a major genetic lineage circulating in mainland China since 1998 [19].

Because immunization is considered the best strategy to control infectious diseases, many vaccines for HFMD are being developed in Asia. For instance, one candidate vaccine is entering phase I clinical trials in Taiwan and Singapore, whereas three candidate vaccines are currently in phase III clinical trials in mainland China [20]. The early clinical trial results suggest that these vaccines are safe and exhibit good immunogenicity when sera from immunized volunteers were tested against EV71 strains of the same subgenotype *in vitro*. However, gene mutations and recombination occur frequently during EV71 epidemics. Therefore, the cross-protection against different subgenotypes induced by immunization in humans is an important practical issue for EV71 vaccine development [21]. As the neutralizing antibody (NtAb) response is a major indicator of EV71 infection and protective immunity, it is used to evaluate the efficacy of HFMD vaccines. The NtAb cross-reactivity among different EV71 strains has not been consistently reported, and the neutralization profiles do not include all EV71 subgenotypes [22], [23].

Whether NtAbs induced by a specific vaccine strain can cross-protect against infections with different subgenotypes of EV71 and which type of vaccine would best match the circulating strains remain unresolved questions. To address these issues, we collected sera from EV71-infected children and vaccine-inoculated individuals (ClinicalTrials.gov identifier: NCT01636245) to measure the cross-reactive NtAb titers against different EV71 subgenotypes using a neutralization assay based on EV71 pseudoviruses [24]. Our results suggest that an inactivated EV71 vaccine derived from EV71-C4a can induce broad cross-neutralizing activity against different EV71 subgenotypes. This study also demonstrates that the new neutralization assay is practical for analyzing clinical samples from epidemiology studies and evaluating vaccines.

## Materials and Methods

### Cells, human sera and virus

293T cells (SV40-transfected human embryonic kidney 293 cells) and RD cells (human rhabdomyosarcoma cells) were cultured as monolayers in Dulbecco's modified Eagle's medium (DMEM, Sigma) supplemented with 10% fetal calf serum (FCS, TBD Co., Ltd, Tianjin, China) (10% FCS–DMEM). 293S cells stably expressing SCARB2, a receptor of EV71 in 293 cells (established by our laboratory [25]) were cultured as monolayers in 10% FCS–DMEM supplemented with puromycin (1.25 µg/ml, Clontech). 293T (ATCC, #CRL-3216), 293 (ATCC, #CRL-1573) and RD (ATCC, #CCL-136) cells were purchased from the American Type Culture Collection (ATCC). 293T cells were used for preparation of the pseudoviruses, while 293S cells were used for pseudovirus titration and the measurement of NtAb titers. RD cells were used to test cytopathic effects (CPE).

Human sera were collected from children aged <5 years at the First Hospital of Jilin University and the Henan Provincial Center for Disease Control and Prevention. Another group of samples was collected from 30 individuals who took part in a phase III clinical study of an inactivated EV71 vaccine (H07, C4a:HQ328793) from Sinovac Biotech Company (ClinicalTrials.gov identifier: NCT01636245). These sera were used to measure the cross-reactive NtAb titers against different EV71 subgenotypes. Approvals were obtained from the Ethics Committee of the Jiangsu Provincial Center for Disease Prevention and Control as well as the Center for Disease Control and Prevention of the Henan province and the First Hospital of Jilin University. Written informed consent was obtained from the parents of all children involved in our study, as well as from the participants.

Live EV71 viruses of different subgenotypes were provided by the National Institute of Diagnostics and Vaccine Development in Infectious Disease, School of Life Science, Xiamen University, China (strain JS52-3 of subgenotypes C4a, strain TW-2006–02203 of subgenotypes B4, strain XMCDC-5535 of subgenotypes B5, strain TW-2008-02969 of subgenotypes C5 and strain TW-2008-03149 of subgenotypes C2) [21].

### Construction of plasmids

**Figure 1:**
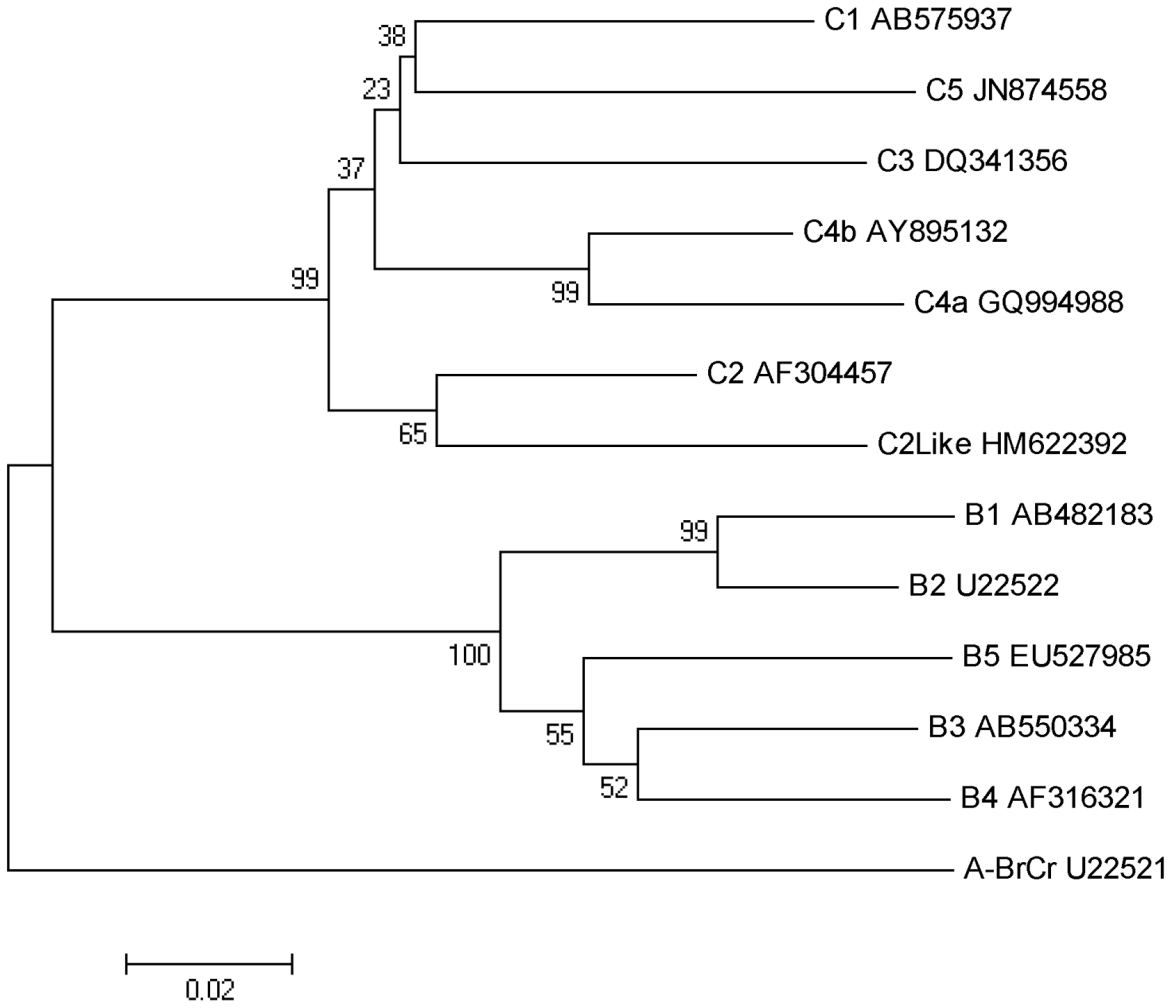
Phylogenetic tree of EV71 subgenotypes. The phylogenetic tree was constructed by the neighbor-joining method with 1000 bootstraps based on nucleotide sequences of EV71 VP1 (891 bp) obtained from GenBank and maximum composite likelihood as the model. EV71-A (BrCr) was used as the outlier. The scale represents differences between sequences or the evolutionary distances used to infer the phylogenetic tree. The bootstrap value was indicated. These EV71 P1 genes were used to construct the plasmids that were prepared to generate the pseudoviruses.

The P1 genes of 12 EV71 subgenotypes were selected for the construction of plasmids [10], [26], [27] (GenBank accession numbers are shown in Figure 1. EV71-A was not prepared because its infectivity was very low and it is not an epidemic strain). The vector VR1012–EGFP–2A–P1 contains the enhanced green fluorescent protein (EGFP) and P1 genes with a linker that encodes the 2A proteinase cleavage motif (sequence of cleavage motif: AAGGGCCTGACCACCTAC). The EGFP-2A-P1 sequence was inserted into the VR1012 vector at the *Sal* I and *Not* I sites. The plasmid pcDNA3.1–T7 RNA polymerase was constructed by inserting the T7 RNA polymerase gene (GenBank ID: M38308) into the pcDNA3.1 vector at the *Xba* I and *Bam*H I sites, and pBlueScript–T7–luc–P2–P3 (encoding the firefly luciferase gene in place of the P1 region) was constructed by inserting the T7 promoter-luciferase-P2–P3 sequence into the pBlueScript II SK (+) vector at the *Pvu* II site. All plasmids were commercially synthesized by Shanghai Jierui Bio-Technology Co., Ltd, China [24], and the vectors were also provided by this company. Sequencing was also performed to confirm the constructs.

### Generation of EV71 pseudoviruses of different genotypes

A 70% confluent monolayer of 293T cells in a T75 (Falcon) flask was cotransfected with 5 µg each of pcDNA3.1–T7 RNA polymerase, pBlueScript-T7-luc-P2-P3 and VR1012–EGFP–2A–P1 using 45 µl of Lipofectamine 2000 reagent (Invitrogen) and then incubated at 37°C in 15 ml of 10% FCS–DMEM. The cells were washed, and the medium was replaced with 10% FCS–DMEM 4 h after transfection. The cells were then incubated for 48 h, followed by freezing and thawing three times to release the pseudovirus. After centrifugation at 12,000×*g* for 10 min to remove the cell debris, the supernatant was filtered through a 0.45 µm membrane and stored at −80°C.

### Measuring infectivity of EV71 pseudoviruses of different genotypes in 293S cells

The infectivity of EV71 pseudoviruses was estimated by infecting 293S cells and measuring the luciferase activity, which was expressed in relative light units (RLU). 293S cells were seeded at a density of 4×10^5^ cells per well in a 6-well plate. After incubation for 24 h, cells in each well were infected with EV71 pseudovirus of different genotypes (50 µl) and incubated for 18 h. The supernatant was discarded, and 100 µl of Bright-Glo™ luciferase reagent [Promega (Beijing) Biotech Co., Ltd] was added to each well before incubation for 3 min in the dark at room temperature. Luciferase activity, expressed in RLUs, was determined with the VICTOR X2 Multilabel Plate Reader (PerkinElmer) according to the manufacturer's instructions.

### Pseudovirus-based neutralization assay

The sera were heat-inactivated at 56°C for 30 min. Fifty microliters of each 20-fold-diluted serum sample was diluted serially three-fold, mixed with 50 µl of working virus solution (which could generate 3.0×10^4^ RLU per well in a 96-well plate) and incubated for 1 h. A suspension of 293S cells at a density of 5×10^4^ cells per well in a 96-well plate was then added and incubated for 18 h. After incubation, the supernatant was discarded, and 50 µl of Bright-Glo™ luciferase reagent [Promega (Beijing) Biotech Co., Ltd] was added to each well before incubation for 3 min in the dark at room temperature. Luciferase activity, expressed in RLUs, was determined with the VICTOR X2 Multilabel Plate Reader (PerkinElmer, USA). Eight wells of virus controls and eight wells of cell controls were included in each 96-well plate. A virus-positive well was defined as a luciferase activity value >3× the mean luciferase activity value of the cell control wells. All serum dilutions were tested in triplicate, and the neutralizing titers were read as the highest dilution that completely inhibited viral growth in over 50% of wells. The neutralization assay based on the EV71 pseudovirus was developed by Jinjun et al. [24].

### CPE assay

The CPE assay was used as the “gold standard” method to test neutralizing antibodies induced by a vaccine in serum samples [28]. In brief, serum samples were inactivated at 56°C for 30 min, serially diluted two-fold from the starting dilution of 1∶8, and then mixed with an equal volume of working virus solution containing 200 TCID_50_ (50% of tissue culture infective dose)/well of EV71 (B4, B5, C2, C4a, or C5) at 37°C for 2 h in a 96-well plate. RD cells (1.0×10^5^ cell/ml) were added to the mixture and incubated at 35°C for seven days. The neutralization titer was expressed as the reciprocal of the highest dilution at which over 50% of the wells showed complete inhibition of CPE. The EV71 NtAbs were defined as positive if the NtAb titers were equal to or greater than that in the 1∶8 dilution [21].

### Statistical analysis

The MEGA 4.1 software [29] was used to generate the phylogenetic tree of EV71 subgenotypes, which was constructed by the neighbor-joining method with 1000 bootstraps based on nucleotide sequences of EV71 VP1 (891 bp), and maximum composite likelihood as the model.

NtAb titers were represented by a heatmap, a graphical method for displaying data by using colors to represent numerical values. The clustering algorithm groups related rows and columns together by similarity. The heatmap is often constructed by R, which is a free software environment for statistical computing and graphics.

In this study, a web tool (http://www.hiv.lanl.gov/content/sequence/HEATMAP/heatmap.html) based on principles of R software was used to make a heatmap of log_10_ transformed the NtAb titers.

Curve fitting was performed with Prism 5 software (GraphPad Software, Inc.).

## Results

### Generation of EV71 pseudoviruses expressing firefly luciferase in 293T cells

To obtain pseudoviruses containing the firefly luciferase gene in place of the P1 gene, 293T cells were triple-transfected with pcDNA3.1-T7 RNA polymerase, pBlueScript-T7-luc-P2-P3 and VR1012-EGFP-2A-P1. The VR1012-EGFP-2A-P1 plasmid provided the source of the P1 protein, whereas pcDNA3.1-T7 RNA polymerase provided the source of the T7 RNA polymerase to transcribe the plasmid pBlueScript-T7-luc-P2-P3. The P1 protein-encapsulated replicon particles expressing luciferase were harvested from the cells.

### Measuring infectivity of EV71 pseudoviruses of different subgenotypes in 293S cells

Pseudoviral particles were examined by electron microscopy, and the expression of the pseudovirus proteins was then examined by immunoblotting. The viral genomic RNA was extracted from the supernatants, and RT–PCR was used to confirm the formation of EV71 pseudoviruses containing the luciferase gene. These examinations were based on the method described by Jinjun *et al.* [24]. We used pseudoviruses of different subgenotypes to infect 293S cells and then measured the luciferase activity, which was expressed in RLUs and reflected the infectivity of the pseudoviruses. The RLU is the light intensity determined by the luminometer [30]. Previous studies have used RLUs as an indicator of pseudovirus infectivity and to represent antibody titers indirectly [31]. Thus, we could compare the efficiency of packaging of each strain and determine the titers of the twelve strains (Figure S1).

### Clinical application of pseudovirus-based neutralization assay to patient serum samples

Patient sera were collected from young children diagnosed with HFMD at the First Hospital of Jilin University (assigned to the Changchun group, 2012) or the Henan Provincial Center for Disease Control and Prevention (assigned to the Luoyang group, 2008). All serum samples had detectable NtAb titers against the EV71 pseudoviruses of different subgenotypes.

EV71-C4, which can be divided into subgenotypes C4a and C4b, has been the major epidemic strain in mainland China in recent years and predominantly responsible for the large current outbreaks, rendering it most suitable as a standard strain [19], [32]. Therefore, we measured the NtAb titers against C4a in four-fold dilutions of all serum samples in the primary screen with the neutralization assay. A positive serum was defined as having a NtAb titer greater than that of the 1∶20 dilution (data not shown). The EV71-positive sera were then selected to further determine their NtAb titers against EV71 pseudoviruses of different subgenotypes in eight-fold dilutions in the secondary screen.

**Figure 2:**
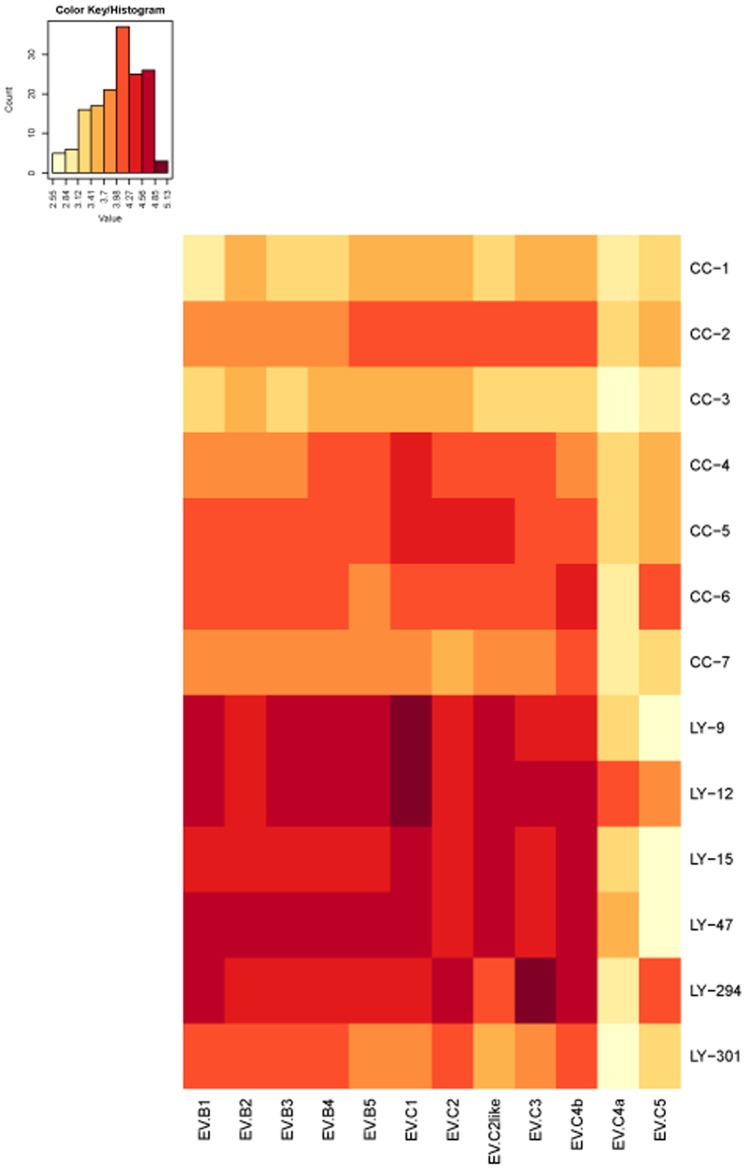
Heatmap analysis of NtAb titers of Changchun and Luoyang samples against EV71 pseudoviruses of different subgenotypes measured in a neutralization assay. Thirteen positive serum samples were identified from the Changchun (CC) and Luoyang (LY) groups. The NtAb titers are shown in yellow for low, orange for middle and red for high titers. The heatmap was generated by a web tool (http://www.hiv.lanl.gov/content/sequence/HEATMAP/heatmap.html). NtAb titers against EV71 pseudoviruses of different subgenotypes, showing the cross-reactivity of the patients' sera, are indicated in the heatmap.

Thirteen positive samples were identified and divided into two groups according to the source of the sera: Changchun (CC) and Luoyang (LY). The serum antibody titers against the different EV71 pseudovirus subgenotypes obtained from neutralization antibody assays of the Changchun and Luoyang groups were log_10_ transformed and then used to construct a heatmap (Figure 2). In the heatmap, color groups represent the NtAb titers. The color scale, which ranges from yellow to red, corresponds with low to high values. The results showed different levels of cross-reactivity of the patient sera with different EV71 subgenotypes.

Results of a secondary screen with the neutralization assay (Figure 2) showed that the general NtAb titers of the Changchun group were lower than those of the Luoyang group against the same pseudovirus subgenotype. However, trends in the NtAb profiles against the different EV71 subgenotypes were similar in the Changchun and Luoyang groups. For instance, the serum NtAb titers against EV71-B4, B5, C1, C2 and C4b were always higher than those against other EV71 subgenotypes. Interestingly, serum NtAb titers against EV71-C4a, which was chosen as the standard strain in the primary screen neutralization assay, were always lower than those against other subgenotypes.

### Clinical application of pseudovirus-based neutralization assay to vaccine-inoculated serum samples

After testing sera of naturally infected patients above, we examined the NtAb cross-reactivity in the context of artificially-induced immunity using sera from 30 vaccine-inoculated individuals who took part in phase III clinical studies of an inactivated EV71 vaccine from Sinovac Biotech Company (ClinicalTrials.gov identifier: NCT01636245). The EV71 strain used (strain H07) belonged to the C4a subgenotype, and the procedure was carried out as described above. First, we measured the NtAb titers against EV71-C4a of all 30 samples in a preliminary experiment, and the 30 samples were divided into three groups according to the relative level of NtAb titer against EV71-C4a: low (L), middle (M) and high (H). NtAb titers against the different EV71 subgenotypes in these vaccine sera were measured in eight-fold dilutions in a secondary screen.

**Figure 3:**
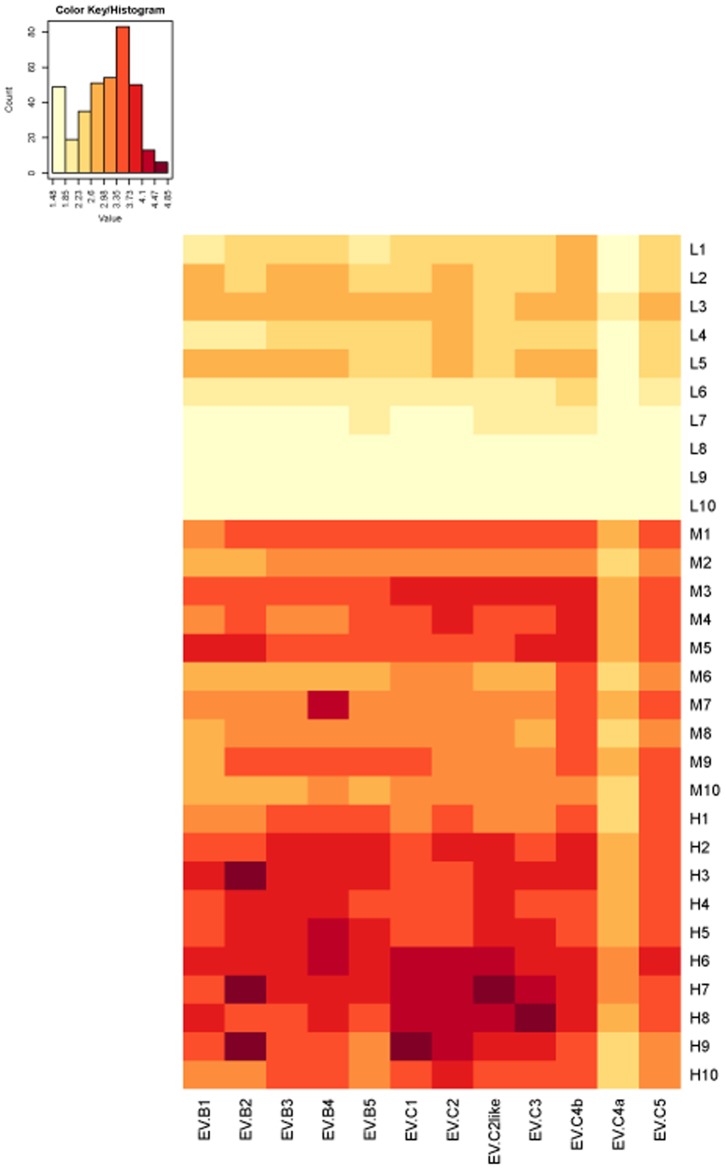
Heatmap analysis of serum antibody titers against EV71 pseudoviruses of different subgenotypes in vaccine-inoculated serum samples measured in a neutralization assay. Thirty samples were divided into three groups according to the relative level of NtAb titer: low (L), middle (M) and high (H). The NtAb titers are shown in yellow for low, orange for middle and red for high titers. The web tool used to generate the heatmap is the same as that used for Figure 2. NtAb titers against EV71 pseudoviruses of different subgenotypes, showing the cross-reactivity of vaccine-inoculated sera, are illustrated in the heatmap.

NtAb titers against EV71 pseudoviruses of different subgenotypes were log_10_ transformed, and a heatmap was constructed based on results of the neutralization antibody assay of the vaccine serum samples (Figure 3). In the heatmap, color groups represent the NtAb titers. These results showed varying levels of cross-reactivity of the vaccine-inoculated sera for the different EV71 subgenotypes. In the low-titer group, NtAb titers of L8, L9 and L10 against the different subgenotypes of the EV71 pseudovirus were all low; therefore, we defined these subgroups as negative controls. Although NtAb titers against the different EV71 subgenotypes differed, some similarities were observed in the trends in the cross-reactive NtAb titers of the three groups (low, middle and high) against the different EV71 subgenotypes. The titers against EV71-B4, B5, C1, C2 and C4b were always higher than those against the other EV71 subgenotypes. The NtAb titers against EV71-C4a were almost always the lowest against all the subgenotypes. These observations were similar to those of the neutralization assay with the Changchun and Luoyang patient samples described above.

### Correlation analysis of the efficacy of pseudovirus and CPE assays in measuring EV71 vaccine-inoculated serum samples

**Figure 4:**
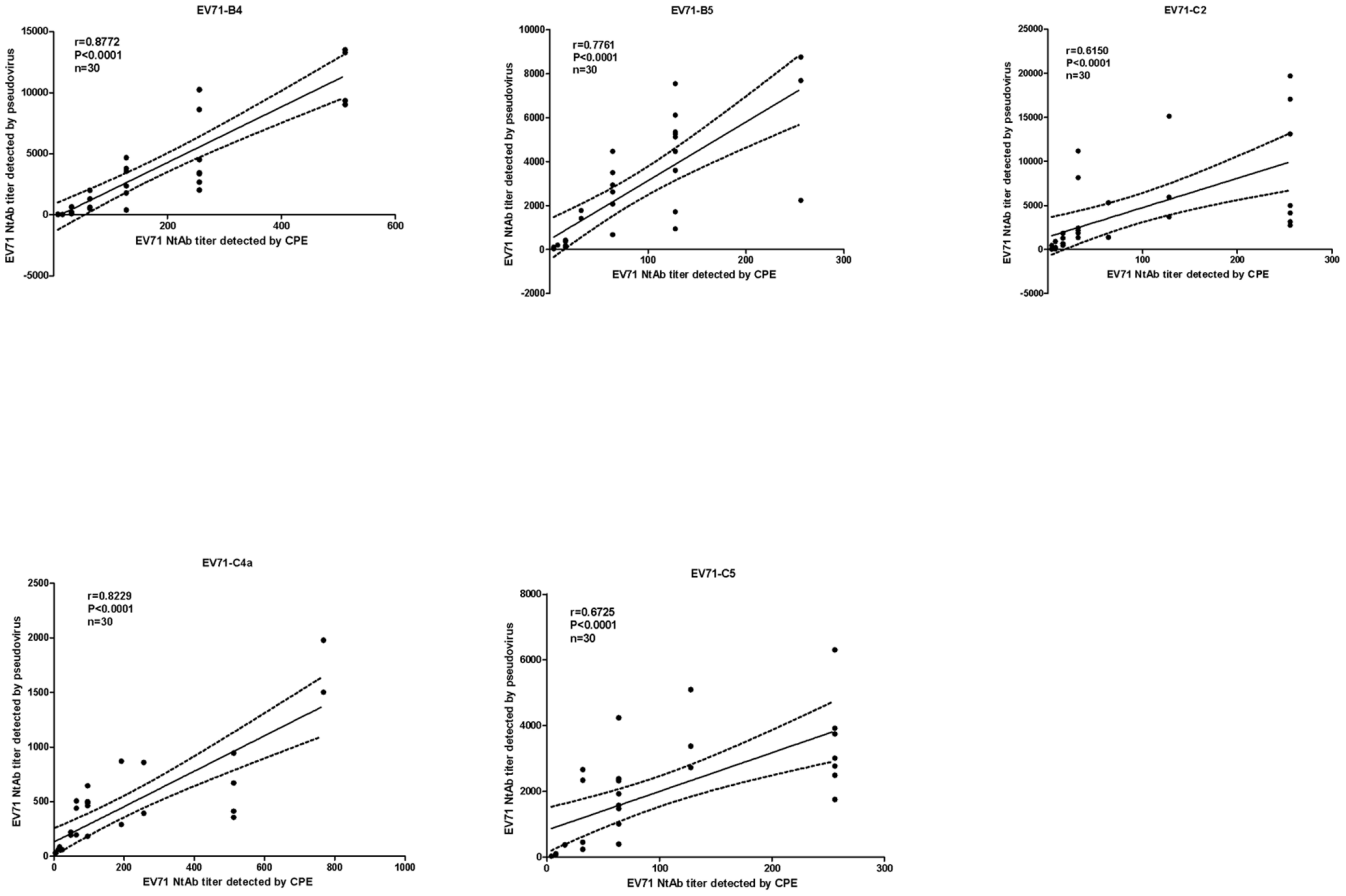
Correlation analyses of NtAb titers against B4, B5, C2, C4a and C5 determined with CPE and pseudovirus assays. The dots represent NtAb titers determined with the CPE and pseudovirus assays. Dotted lines represent the 95% confidence band, and solid lines were fitted from the NtAb titers measured by the two methods. NtAb titers against B4, B5, C2, C4a and C5, quantified with the pseudovirus assay, showed a statistically significant correlation with those detected with the CPE assay (Pearson's r≥0.6150, *P*<0.0001 in all cases).

The CPE assay is a traditional method of measuring NtAb titers of serum samples. To determine the efficacy of the pseudovirus-based assay, we measured NtAb titers of the EV71-C4a vaccine-inoculated sera from a phase III trial (ClinicalTrials.gov identifier: NCT01636245) with the CPE method and performed a correlation analysis of the results of all 30 samples tested with those of the pseudovirus assay using EV71-B4, B5, C2, C4a and C5. Higher NtAb titers were found in the pseudovirus assay than in the CPE assay for every subgenotype of EV71. However, Pearson's r values between the two assays were 0.8772, 0.7761, 0.6150, 0.8229 and 0.6725 for the B4, B5, C2, C4a and C5 subgenotypes, respectively. Thus, results of these two assays were significantly correlated for each subgenotype of EV71 (Pearson's r ≥0.6150, *P*<0.0001 in all cases; Figure 4). While the new pseudovirus-based method exhibited a high degree of agreement with the CPE assay, it was more sensitive, consistent with our previous study [21], [24], [33].

## Discussion

Although EV71 has one serotype, it has multiple subgenotypes and consequently varied antigens. Therefore, the selection of a vaccine strain that can induce broad cross-protective NtAbs is particularly important for the development of an effective EV71 vaccine. EV71 NtAbs are critical for monitoring an EV71 epidemic and evaluating the immunogenicity of a vaccine. While raising effective cross-reactive NtAbs using a vaccine against viruses with high genetic variability, such as HIV [34]–[36] and influenza [37], can be difficult, some studies have indicated that eliciting NtAbs against one subgenotype of EV71 is likely to confer cross-neutralization against certain other subgenotypes [21]–[23], [38], [39]. Several such neutralization studies were conducted using animal models. Minetaro *et al.* characterized the antigenicity of an attenuated strain of EV71-A (BrCr) in a monkey infection model and measured the NtAbs against EV71-A, B1, B4, C2 and C4. Levels of NtAb titers elicited were ordered as follows: A>B1>C4>B4>C2 [22]. Mizuta *et al.* analyzed the cross-neutralization capacity of sera from guinea pigs that had been immunized with the B2 and C1 strains. Their study showed differences in the serum NtAb titers against the six subgenotypes (B2, B4, B5, C1, C2 and C4) and suggested that genogroup C strains are more difficult to neutralize than genogroup B strains or that genogroup B strains readily induce higher NtAb titers than genogroup C strains [23]. As another example of the use of a neutralization assay with naturally infected human sera, Huang *et al.* examined the cross-neutralization capacity of sera from 25 young Taiwanese children who were infected with EV71 subgenotypes C2, B4, C4, B5 or C4 from 1998–2010. They reported that children infected with genotype C had similar NtAb titers against genogroups B and C, whereas children infected with genotype B had higher NtAb titers against genogroup B than those against genogroup C. These results indicated that humans naturally infected with EV71 genotype C could generate serum antibodies with high cross-protective activity against other EV71 strains [39]. Recently, several neutralization studies with EV71 vaccine-inoculated human sera have been reported. Chou *et al.* demonstrated that cross-neutralizing antibody responses could be elicited in volunteers treated with a B4 strain EV71 vaccine against several different EV71 subgenotypes (B1, B4, B5, C2, C4a and C4b). The results indicated that an EV71 vaccine based on the subgenotype B4 strain would be a good candidate vaccine, although the NtAbs it raised did not neutralize the C2 strain [38]. Mao *et al.* collected serum samples from a subset of 119 participants (aged six months to 11 years) in two clinical trials of an inactivated EV71 (subgenotype C4a) vaccine to detect their NtAb titers. A CPE method was used to assess the NtAbs against B4, B5, C2, C4a and C5 subgenotype strains of EV71, which have been prominent epidemic strains worldwide over the past decade. The results suggest that the inactivated EV71 vaccine derived from subgenotype C4a induced broad cross-neutralizing activity in Chinese infants and children [21]. However, results of the studies described above were not consistent across different experimental models (animal and human) and immunogens, and the findings were not relevant to all EV71 subgenotypes. Since different laboratory procedures, cell lines and virus strains used in the neutralization assay would introduce variations in results of different studies, it is important to establish an international standard protocol for measuring NtAbs.

Several studies have examined the cross-neutralization potential of immunized animal sera and detected some level of cross-reactivity against several different EV71 strains. However, the cross-protective capacities in these animal studies differed greatly, and the findings often were conflicting. Therefore, determining which animal model (e.g., rabbit, mouse or macaque) that would be most suitable to conduct immunogenicity studies is important for evaluating the potency of EV71 vaccines [22], [23]. Few studies have focused on the cross-neutralizing activity of vaccinated human sera, especially those of infants and children, who are in the target population [21], [38]. In this study, we chose the sera of patients inoculated with an EV71 vaccine (EV71-H07, C4a strain) in a clinical trial to detect NtAbs and to evaluate the cross-neutralizing immunity elicited by vaccination with a single subgenotype of EV71.

EV71-induced immunogenicity and the prevalence of EV71 infection in epidemiological studies are evaluated traditionally with CPE-based methods [28]. However, CPE assays are labor-intensive, subjective and time-consuming, making them unsuitable for measuring large numbers of samples and assessing a whole panel of various EV71 subgenotypes. In this study, we applied a new pseudovirus-based method to resolve these issues. Pseudoviruses of different EV71 subgenotypes were constructed to detect NtAb titers of serum samples from different sources with this neutralization assay. However, our library of pseudoviruses contained only the EV71-B and C groups, but not the EV71-A group (BrCr). Due to a technical difficulty, pseudoviruses of the A group with sufficiently high infectivity could not be obtained. EV71-A (BrCr) was identified in 1970 in the USA, but it was not detected globally again until 2008 [11]. In an HFMD outbreak in China in 2008, Yu *et al.* identified five EV71 isolates that were closely related to genotype A based on an analysis of their VP1 genes. However, these genogroup A viruses did not spread widely, and the reason for the reemergence of genotype A in China is unclear [40]. Because EV71-A is not a major epidemic strain globally, our results still support that C4a strain can elicit broadly NtAbs. In addition, the EV71 C2-like genotype is a unique new subgenogroup of EV71 that was identified in Taiwan in 2008. Interestingly, this novel C2-like virus was a recombinant of C2 and B3 subgenotype viruses, but it did not spread widely [10]. In our study, we also selected a C2-like strain as one subgenotype from the library for detection of broad cross-reactivity. Another important subgenotype is EV71-C4, a major epidemic strain in mainland China in recent years, which can be divided into the C4b and C4a evolutionary branches that correspond to two time periods. The C4b evolutionary branch contains strains from Shenzhen and Shanghai from 1998–2004, and C4a has been found in mainland China since 2003. The clade transition suggests that the evolution of EV71 subgenotype C4 occurred during its persistent circulation in mainland China within a decade [19], [32].

In measuring the NtAb cross-reactivity of sera from patients who were naturally infected with EV71, we found that trends in the NtAb titers against different EV71 subgenotypes were similar in the Changchun and Luoyang serum samples (Figure 2). Subgenotype C4a was used as the standard strain in the first round of neutralization assays to identify the positive sera, and then pseudoviruses based on different EV71 subgenotypes were employed in the second round of neutralization assays to measure the cross-reactivity of the NtAbs. Almost all of the positive sera were found to have higher NtAb titers against genogroup B and subgenotypes C1, C2 and C3 than against subgenotype C4a. Such cross-reactive NtAb responses are important for vaccine development. Because serum samples were collected from young children (aged <5 years) in Changchun (northeastern region of China, 2012) and Luoyang (Central Plains region of China, 2008), and EV71-C4a was the major epidemic strain in China in recent years [19], [41], [42], the results suggest that the C4a subgenotype could induce cross-reactive NtAbs against different EV71 subgenotypes in these patients. In screening of the patients' sera with the neutralization assay, we also found that trends in the NtAb titers of Luoyang-294 and -301 against EV71 pseudoviruses of different subgenotypes were more similar to those of the Changchun samples, but different from those of other Luoyang samples. This phenomenon may reflect the evolution of the EV71 virus over time (2008–2012) and variations in subgenotype C4 in different regions of China (Luoyang and Changchun).

We also analyzed the NtAb titers of vaccine-inoculated sera against EV71 pseudoviruses of different subgenotypes. Trends in the NtAb titers against the different EV71 subgenotypes were similar in the low-, middle- and high-titer groups (Figure 3). The inactivated EV71 vaccine was derived from subgenotype C4a, and the NtAb titers of each titer group (low-, middle- and high-titer groups) tended to be similar. Therefore, the cross-reactivity of the sera against EV71 pseudoviruses of different subgenotypes reflected the cross-protective effects of NtAbs induced by the C4a vaccine against different EV71 subgenotypes. This broad cross-neutralizing capacity of the serum EV71 NtAbs induced by the C4a vaccine was similar to that induced by natural infection [21]. Together, the results indicate that the C4a subgenotype virus would be a good candidate EV71 vaccine strain for mainland China.

Clinical trials of an inactivated EV71 vaccine suggested that it had a clinically acceptable safety profile and good immunogenicity in healthy Chinese infants and children [21], [43], [44], but the NtAb titer against C4a was lower than that against other subgenotypes. In our study, NtAbs induced by EV71 C4a were cross-reactive against different EV71 subgenotypes. Importantly, the results suggest that the EV71-C4a strain vaccine may provide cross-protection against almost every EV71 subgenotype in active circulation worldwide, especially in China, because NtAbs titers induced by this strain were all higher than 1∶32, which has been suggested as a surrogate of protection against EV71-associated disease [20]. Thus, all the samples could be defined as NtAb positive. However, NtAb titers against C4a were almost always the lowest of all the EV71 subgenotypes in our study, indicating that the current genotyping of EV71 does not reflect their antigenicity, and this conclusion has been was discussed by other researchers [2], [10], [45]. Both the EV71 vaccine strain (H07, HQ328793) and the standard detection pesudovirus (GQ994988) used in this study were C4 epidemic strains in China and were classified as C4a, one of two branches of C4 (C4a and C4b). We also constructed other C4 epidemic strains for further study. Our tests for cross-reactive NtAbs in sera derived from mice inoculated by an inactivated EV71 vaccine or virus-like-particle (VLP) (C4:EU703814) vaccine also demonstrated that the NtAb induced by one C4 strain could be cross-reactive against other C4 strains and EV71 pseudoviruses of different genotypes. Some of the results had been included in our another paper (submitted, data not shown). Results of the CPE assay demonstrated that the NtAb titers induced by C4a (HQ328793) against C4a were also lower than those against B4, B5, C2 and C5, similar to findings of a prior study [21]. The phenomenon that NtAbs induced by C4 showed poor efficiency against EV71-C4 is difficult to explain. These results may be related to the specific cell line and viruses strains used, and the capsid structure also could have influenced the antigenicity of the virus.

In comparing the pseudovirus-based neutralization assay with the CPE-based assay for evaluating the cross-neutralizing activities of serum samples in this study, EV71 strains with subgenotypes B4, B5, C2, C4a and C5 were selected. C2 and C4 were the predominant strains responsible for HFMD in recent years, whereas B4, B5 and C5 were the prominent strains in the past decade; therefore, these five subgenotypes would be the most representative for determining the cross-protective capacity of the EV71 vaccine [21]. A good correlation was found between the CPE-based assay with the pseudovirus-based assay when measuring NtAb titers of vaccine-inoculated sera against the different EV71 subgenotypes (B4, B5, C2 C4a, and C5), which supports the reliability of results in this study. These results were also consistent with our previous studies [21], [24], [33].

Coxsackievirus A16 (CVA16) is another causative pathogen of HFMD in China [46]. Since CVA16-associated HFMD is usually mild and benign, more interest has been placed on EV71. However, many recent reports showed that CA16 infections may also lead to severe health issues [47]. Therefore, CVA16 and the development of a CVA16 vaccine has received increasing attention [48]. Zhao *et al*. found that circulating CVA16 strains in China were recombinant viruses involving multiple HEV-A strains, including CVA4, CVA16 and EV71, but were not quite related to the prototype CVA16-G10 in different parts of the viral genome [49]. Their study characterized the full-length genome of circulating CVA16, which would facilitate the development of CVA16 vaccines to control this disease. In future studies, we will also construct CVA16 pseudoviruses of different genotypes to detect NtAb titers against CVA16 strains, which would allow broader evaluations of different CVA16 vaccines.

In the present study, we detected the cross-reactivity of sera from naturally-infected patients and then measured sera derived from volunteers inoculated with an inactivated EV71-C4a vaccine as part of phase III clinical studies. The new pseudovirus-based assay for the quantitative measurement of serum anti-EV71 NtAbs was also validated by comparing it against a CPE-based method. The results demonstrated that EV71 vaccination with subgenotype C4a, which is prevalent in mainland China, could provide broad cross-protection against different EV71 subgenotypes in healthy children and infants. However, due to variations and recombination of different EV71 subgenotypes over time, monitoring EV71 epidemics at different times and places remains a high priority in order to ensure that the disease is controlled and effective vaccines are developed accordingly. In future studies, we will construct pseudoviruses based on additional epidemic strains, including other EV71-C4 strains, to further characterize the antigenicity and genetic evolution of EV71. These expanded efforts will allow pseudovirus-based neutralization assays to be more widely used in analyses of clinical samples and evaluations of different types of EV71 vaccines.

## Supporting Information

Figure S1 **Infectivity of EV71 pseudoviruses of different genotypes.** Luciferase activity, expressed in RLUs, reflect the infectivity of EV71 pseudoviruses of different genotypes. (C2L is the abbreviation for C2-like).
(TIF)
