# Supplementary figures and images for: Analysis of Cross-Reactive Neutralizing Antibodies in Human HFMD Serum with an EV71 Pseudovirus-Based Assay
Source: PLoS One. 2014 Jun 25;9(6):e100545. doi: 10.1371/journal.pone.0100545 (PMC4070950; doi:10.1371/journal.pone.0100545)

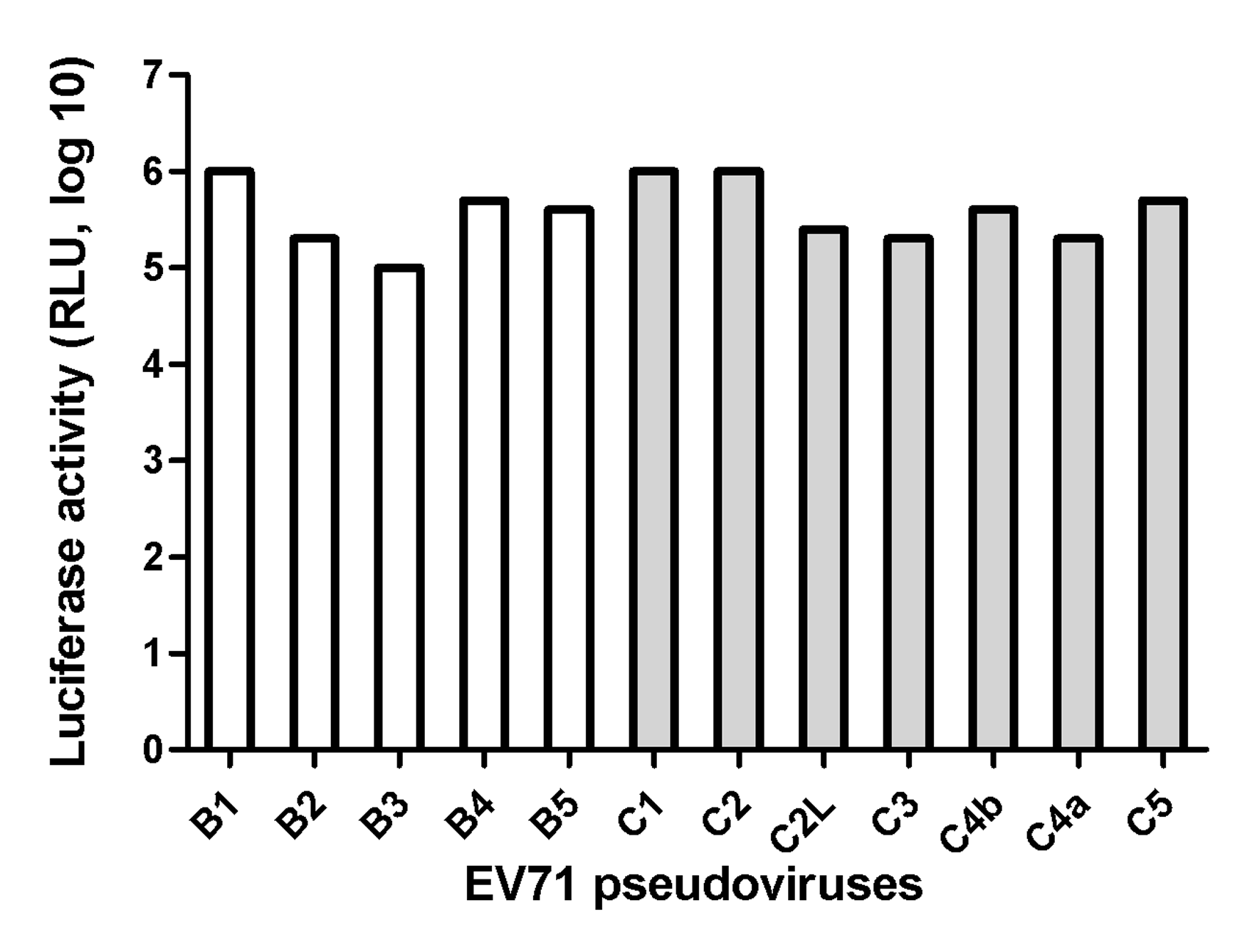

Supplement: Figure S1 — Infectivity of EV71 pseudoviruses of different genotypes. Luciferase activity, expressed in RLUs, reflect the infectivity of EV71 pseudoviruses of different genotypes. (C2L is the abbreviation for C2-like). (TIF) [file pone.0100545.s001.tif]
